# American Journal of Medical Sciences

**Published:** 1861-08

**Authors:** 


The most important feature of the current number of Dr. Hay's *Journal of Medical Sciences*, is the valuable paper of Dr. W. W. Morland,—"*The Morbid Effects of the Retention in the Blood of the Elements of the Urinary Secretion*,"—the Fisk Fund Prize Essay. The review and bibliographical departments are full and interesting; and the summaries of foreign and domestic intelligence, as usual, comprehensive.

Of our other exchanges, our readers are able to judge for themselves, from the numerous excerpta we make from time to time; though occasionally, as in the present number, a pressure of original matter abridges the space usually occupied by selections.

